# Meniscal tears are more common than previously identified, however, less than a quarter of people with a tear undergo arthroscopy

**DOI:** 10.1007/s00167-021-06458-2

**Published:** 2021-02-01

**Authors:** Imran Ahmed, Anand Radhakrishnan, Chetan Khatri, Sophie Staniszewska, Charles Hutchinson, Nicholas Parsons, Andrew Price, Andrew Metcalfe

**Affiliations:** Warwick Clinical Trials Unit, Clinical Sciences Research Laboratories, University Hospital Coventry and Warwickshire, Clifford Bridge Road, Coventry, CV22DX UK

**Keywords:** Meniscal tears, Arthroscopy, Knee pain, MRI

## Abstract

**Purpose:**

The management of meniscal tears is a widely researched and evolving field. Previous studies reporting the incidence of meniscal tears are outdated and not representative of current practice. The aim of this study was to report the current incidence of MRI confirmed meniscal tears in patients with a symptomatic knee and the current intervention rate in a large NHS trust.

**Methods:**

Radiology reports from 13,358 consecutive magnetic resonance imaging scans between 2015 and 2017, performed at a large UK hospital serving a population of 470,000, were assessed to identify patients with meniscal tears. The hospital database was interrogated to explore the subsequent treatment undertaken by the patient. A linear regression model was used to identify if any factors predicted subsequent arthroscopy.

**Results:**

1737 patients with isolated meniscal tears were identified in patients undergoing an MRI for knee pain, suggesting a rate of 222 MRI confirmed tears per 100,000 of the population aged 18 to 55 years old. 47% attended outpatient appointments and 22% underwent arthroscopy. Root tears [odds ratio (95% CI) 2.24 (1.0, 4.49); *p* = 0.049] and bucket handle tears were significantly associated with subsequent surgery, with no difference between the other types of tears. The presence of chondral changes did not significantly affect the rate of surgery [0.81 (0.60, 1.08); n.s].

**Conclusion:**

Meniscal tears were found to be more common than previously described. However, less than half present to secondary care and only 22% undergo arthroscopy. These findings should inform future study design and recruitment strategies. In agreement with previous literature, bucket handle tears and root tears were significant predictors of subsequent surgery.

**Level of evidence:**

III.

**Supplementary Information:**

The online version contains supplementary material available at 10.1007/s00167-021-06458-2.

## Introduction

Meniscal tears have been estimated to affect 60–70 per 100,000 of the population [1]. They can affect younger patients with higher functional demand as well as older patients who may have pre-existing degeneration in the knee [2, 3]. Both the British Association for Surgery of the Knee (BASK) and the European Society for Sports Traumatology, Knee Surgery and Arthroscopy (ESSKA) have produced consensus guidelines on the treatment for meniscal tears [4–6]. This identifies clinical scenarios where urgent surgery is indicated, surgery after a period of physiotherapy is indicated and no surgery is indicated. The guidelines recommend the need for an initial period of conservative management (where urgent surgery is not indicated) followed by further investigations to rule out osteoarthritis before considering surgery [5]. Of particular interest in the guidelines is the identification of specific meniscal ‘target lesions’. These are specific tear patterns where surgery may be indicated. Treatment decisions for certain tear types such as bucket handle or root tears are clear in the guidelines, however, for other tear patterns they are less clear. Further work is needed to identify the incidence of each type of target lesion diagnosed on magnetic resonance imaging (MRI) and the incidence of degenerative changes in patients with a meniscal tear. Research has shown 60–90% of patients with osteoarthritis have a meniscal tear [7], however, work is needed to identify how many patients with a meniscal tear also possess degenerative changes.

Recent studies have highlighted the need for research to identify a specific subset of patients where surgery may be beneficial and to undertake a study in this population [8–11]. However, before this work can be carried out more evidence is required to understand the current population of patients with meniscal tears. Research is required to identify the current incidence of MRI confirmed tears, the incidence of each type of meniscal target lesion and the current intervention rate in current clinical practice.

There is also a need to understand any patterns in referral to secondary care. Once in secondary care, further understanding is needed to identify whether any factors strongly influence the need to undergo operative management.

The aim of this study is to review consecutive MRI scans performed for patients with a symptomatic knee over a 3-year period at a large NHS trust to:

1. Explore the epidemiology of meniscal tears, in particular, to identify the current incidence of MRI confirmed meniscal tears in patients with a symptomatic knee in current practice and the proportion of these that undergo surgical management.
2. Describe the incidence of each type of meniscal target lesion and the proportion of these which also have degenerative changes within the knee.
3. Explore the association between meniscal target lesions and arthroscopic surgery in current practice in a large NHS trust.

The study hypothesis is that meniscal tears are more common than previously reported with the majority being identified in primary care. The secondary hypothesis is that bucket handle tears and root tears will have higher surgery rates than other tear types.

## Materials and methods

The study, which used routinely collected anonymised data only, was registered at the UHCW research and development team as a service evaluation project (ref. SE0168). In addition, ethical approval was granted by the University of Warwick Biomedical and Scientific Research Ethics Committee (ref. BSREC 09/19-20) on the 11th October 2019. This study is reported in accordance with the Strengthening The Reporting of Observational studies in Epidemiology (STROBE) Guidelines for Observational Studies and Statistical Analyses and Methods in the Published Literature guidelines [12].

A single-centre retrospective study was undertaken at University Hospital Coventry and Warwickshire NHS trust, which includes both University Hospital Coventry and Rugby St. Cross hospital. All consecutive MRI knee scans taken between 1st January 2015 and 31st December 2017 at the two hospitals were reviewed against the following eligibility criteria:

Inclusion criteria:

- Meniscal tear of any type caused by any mechanism
- MRI written report available
- Patient aged between 18 and 55 years
  Exclusion criteria:
- Additional ligament rupture.
- Fracture of the tibial plateau or femoral condyle.
- Septic arthritis or infection within the knee.
- Previous knee surgery.
- Previous meniscal tear in the same knee.

MRI scans up to 2017 were included to ensure at least 2 years of treatment data from the date the scan was available for analysis.

Using procedure codes, all MRI knee reports over a 3-year period were reviewed. Three authors (IA, AR, CK) reviewed the available MRI reports to identify the patients which met the eligibility criteria above. One author (IA) then cross checked all the reports to ensure accuracy of screening and patient identification. From the MRI report, the following data were collected: patient age, sex, right/left knee, tear location (medial/lateral), tear type referral source (GP/outpatients/emergency department), displaced/undisplaced, presence of chondral changes and whether the report recommended an orthopaedic referral. Chondral changes were identified if the report included the following terms: tricompartmental arthritis, osteoarthritis within the knee, chondral thinning or loss. Electronic hospital records were then interrogated to collect the number of orthopaedic outpatient appointments the patient attended and any surgical procedures. The outcomes of interest for this study were:

- The rate of meniscal tears in the current population.
- The proportion of patients with a meniscal tear undergoing arthroscopic surgery.

### Statistical analysis

Patient characteristics were summarised by calculating means and standard deviations for continuous outcomes (e.g. age) and tabulations to show rates for categorical outcomes (e.g. sex, tear location). To explore the relationship between the categorical data and the primary outcome (incidence of arthroscopic knee surgery) chi-squared tests were calculated, with significance set at the 5% level. A logistic regression analysis was used to further explore the relationship between tear type and surgery. First, the data were limited to patients who had a unilateral tear in one knee. The reason for this was to ensure that the outcome of interest was related to that specific type of meniscal tear. For the primary outcome, the response variable in the linear regression was the incidence of arthroscopic surgery and the explanatory variables were age, sex, tear type, tear location, chondral changes, presence of root tears and size description. A stepwise procedure, with forwards selection and backwards elimination, was used to identify which variables were significant in the final model; statistical significance was assessed at the 5% level. The odds ratios between each dependent variable and arthroscopy from the final fitted model were used to draw inferences on the strength of associations, with graphical plots created where appropriate. A similar method was employed for the secondary outcome (number of outpatient appointments), however, rather than a linear regression model a Poisson regression model was used, to account for the fact that these were counts. All analysis was carried out using R (R Core team (2013) R Foundation for statistical computing, Vienna, Austria) [13].

## Results

Between January 2015 and December 2017, there were 13,358 MRI knee scans performed at University Hospital Coventry and Warwickshire (UHCW) NHS trust. From this, 8868 were performed in 18 to 55-year-old patients and 1737 (13%) had an isolated meniscal tear.

The mean age of patients found to have a meniscal tear was 43.1 years. There was a greater number of meniscal tears in males (*n* = 1059; 61%) compared to females (*n* = 678; 39%). The majority (1330; 76.6%) of MRI scans which had a new meniscal tear were requested by a general practitioner (GP, a primary care doctor), and the minority by a clinician in a secondary care setting (377; 21.7%).

The most common type of tears as described by the radiologists were horizontal, oblique or undersurface tear (*n* = 577; 42%) followed by complex tears (*n* = 214; 15.5%), degenerative tears (*n* = 208; 15.1%), radial (*n* = 187; 13.6%), degenerative horizontal, oblique, undersurface tears (*n* = 101; 7.3%) and bucket handle tears (*n* = 88; 6.4%). Only 49 (2.82%) of MRI reports specifically mentioned the presence of a root tear. Additional descriptive terms were used in the MRI reports provided by the radiologists. 876 (50.4%) reported the presence of chondral changes within the knee. 193 (11.1%) reports described the tear as small or undisplaced and 119 (6.85%) of reports recommended referral to an orthopaedic specialist.

Of the patients with a new isolated meniscal tear, 821 (47.3%) were reviewed in an orthopaedic outpatient clinic. For patients with a handle tear, 52 out 88 (59%) were seen in an outpatient clinic. This proportion of outpatient attendance was higher than patients with a complex [107 out of 214 (50%)], degenerative [101 out of 208 (48.6%)], horizontal/oblique/undersurface [253 out of 577 (43.8%)] and radial tear [81 out of 187 (34.7%)]. Patients with a horizontal/oblique/undersurface had the lowest proportions seen in outpatients [35 out of 101 (34.7%)]. A logistic regression model was fitted to the data, with the result that no variables were found to be statistically significant at the 5% level when using attendance to outpatients as the outcome of interest. Further details of the model results can be seen in Table 1.

**Table 1:** Estimated odds ratios (OR) and 95% confidence intervals for each variable in the logistic regression model for outpatient attendance

Variable	Subgroup	Odds ratio	95% confidence interval	*p*-Value
Gender	Female	1.0		
	Male	0.86	(0.83, 3.13)	n.s
Age groups	18–40	1.0		
	40–50	0.74	(0.57, 0.98)	
	50–55	0.84	(0.62, 1.16)	n.s
Tear type	Bucket handle	1.0		
	Complex	0.80	(0.46, 1.40)	
	Degenerative tear	0.72	(0.41, 1.28)	
	Degenerative horizontal/oblique/undersurface	0.42	(0.22, 0.82)	
	Horizontal/oblique/undersurface	0.64	(0.38, 1.08)	
	Radial	0.60	(0.33, 1.07)	n.s
Tear location	Lateral anterior horn	1.0		
	Lateral anterior horn and body	1.09	(0.44, 2.68)	
	Lateral body	0.81	(0.36, 1.78)	
	Lateral meniscus	1.09	(0.56, 2.12)	
	Lateral posterior horn	1.01	(0.54, 1.88)	
	Lateral posterior horn and body	2.09	(0.82, 5.66)	
	Medial anterior horn	1.40	(0.51, 3.98)	
	Medial body	0.92	(0.47, 1.76)	
	Medial meniscus	1.22	(0.67, 2.24)	
	Medial posterior horn	0.96	(0.64, 1.45)	
	Medial posterior horn and body	0.87	(0.53, 1.43)	n.s
Referral recommendation	Referral not recommended	1.0		
	Referral recommended	0.69	(0.44, 1.05)	n.s
Degeneration	No chondral changes	1.0		
	Chondral changes	1.15	(0.91, 1.47)	n.s
Tear size	Not undisplaced/small	1.0		
	Undisplaced/small	0.78	(0.53, 1.16)	n.s
Root tears	No root tear	1.0		
	Root tear	1.08	(0.53,2.21)	n.s

Of the 1737 patients with a meniscal tear, 378 (21.8%) subsequently underwent arthroscopic surgery at UHCW NHS trust. Chi-squared tests found that males were more likely to have surgery compared to females (*p* = 0.031). There was a statistically significant association between younger patients and arthroscopy olds (chi-squared test, *p* = 0.002). Chondral changes were seen in 870 (50.1%) patients with a meniscal tear. 169 (28.9%) of patients without chondral changes underwent arthroscopy, whereas, 131 (19%) of patients with chondral changes underwent arthroscopy (*p* = 0.011).

For further details on the results of the individual chi-squared tests please see Supplementary Table 1.

**Fig. 1:**
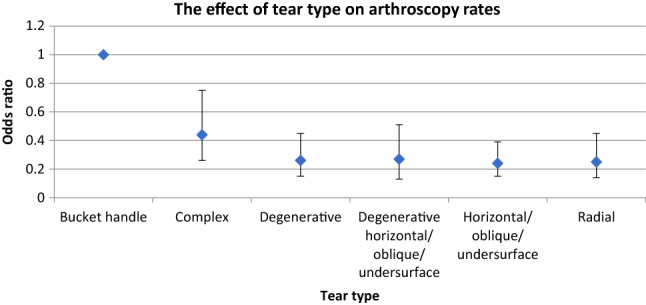
Estimated odds ratios (bars are 95% confidence intervals) from the arthroscopy model for each tear type (odds ratio for bucket handle tears is set to one)

Table 2 provides a summary of the estimates of odds ratios and 95% confidence intervals for the results of the logistic regression model. Bucket handle tears were statistically significantly more likely to undergo arthroscopy than all other tear tears (*p* < 0.001), however, there was no statistically significant difference in OR between the remaining tear types. Figure 1 provides a graphical representation of the difference in ORs of the different tear types. The presence of a root tear also significantly increased the probability of an arthroscopy (OR 2.24 95% CI 1.0, 4.75; *p* = 0.049) (Table 2). The age of patients, presence of chondral changes, the recommendation of referral or tear size had no statistically significant effect on arthroscopy rates (Table 2).

**Table 2:** Estimated odds ratios (with 95% confidence intervals) for model variables from logistic regression model for arthroscopy

Variable	Subgroup	Odds ratio	95% confidence interval	*P*-value
Gender	Female	1.0		
	Male	1.13	(0.85, 1.88)	n.s
Age groups	18–40	1.0		
	40–50	0.87	(0.63, 1.20)	
	50–55	0.66	(0.45, 0.97)	n.s
Tear type	Bucket handle	1.0		
	Complex	0.44	(0.26, 0.75)	
	Degenerative tear	0.26	(0.15, 0.45)	
	Degenerative horizontal/oblique/undersurface	0.27	(0.13, 0.51)	
	Horizontal/oblique/undersurface	0.24	(0.15, 0.39)	
	Radial	0.25	(0.14, 0.45)	< 0.001
Root tear	No root tear	1.0		
	Root tear	2.24	(1.0, 4.75)	0.049
Referral recommendation	Referral not recommended	1.0		
	Referral recommended	0.83	(0.45, 1.37)	n.s
Chondral changes	No chondral changes	1.0		
	Chondral changes	0.81	(0.60, 1.08)	n.s
Tear size	Not undisplaced/small	1.0		
	Undisplaced/small	0.62	(0.35, 1.05)	n.s

## Discussion

The most important findings of the present study are despite meniscal tears being more common than previously reported, less than half of patients with a meniscal tear present to secondary care and only 21.8% undergo arthroscopic surgery. Over a 3-year period between 2015 and 2017, 1737 patients aged between 18 and 55 with a symptomatic knee were diagnosed with a new meniscal tear in a trust which serves a population of 470,430 people [14, 15]. Population-based studies suggest that approximately 261,351 people are aged between 18 and 55 [14, 15]. Based on this study data the rate of new meniscal tears diagnosed on MRI in patients with a symptomatic knee was 222 per 100,000 population.

Previous literature has reported an incidence of meniscal tears of between 60 and 70 per 100,000 [16, 17]; the studies were performed over 20 years ago and used different diagnostic criteria largely based on clinical examination or arthroscopic findings. Explanations for the higher rate in this study include the inclusion of both operatively managed and non-operatively managed patients and the use of MRI to diagnose meniscal tears. The authors believe this estimate is an underestimate of the true rate as the sample was based on patients who underwent an MRI due to knee pain. In previous literature, up to 60% of patients were found to have a meniscal tear with no history of knee pain, although it is questionable if they are clinically relevant or symptomatic [7, 18].

Tear type and presence of root tears were statistically significant predictors of subsequent surgery. This corresponds with the BASK treatment guidelines which suggest certain target lesions may benefit from surgery (if corresponding symptoms exist) [4]. Bucket handle tears had the highest proportion undergoing subsequent surgery (50%). Following this, a complex tear had the second highest OR 0.44 (95% CI 0.26, 0.75). All other tear types had a similar OR (0.24–0.27). It is therefore very difficult to predict the need for subsequent surgery-based solely on tear pattern. This highlights the importance of taking into account other features in the decision-making process including duration and type of symptoms including meniscal specific symptoms such as locking [4, 19]. In addition, the description of a tear being degenerative in appearance does not have any value in terms of subsequent treatment and the authors question the use of this term. A radiologist recommendation that orthopaedic advice should be sought also had no influence on the subsequent need for surgery and the authors do not recommend the use of these terms in future reports.

Previous research has demonstrated that arthroscopy in the context of arthritis may not be beneficial and a numerous of treatment guidelines state arthroscopy for osteoarthritis is not indicated [3–5, 8, 20]. Chondral changes were most commonly found in degenerative or degenerative horizontal/oblique/undersurface tears. A reason for this is that these tears were more common in older patients who are more susceptible to degenerative changes. This study showed that the presence of chondral changes in the context of a meniscal tear did not affect rates of surgery.

One of the main strengths of this study is that it included all MRI reports performed in a large NHS trust over a 3-year period. The trust itself is the only NHS trust serving a region with a population of over 400,000. There are private providers in the region but these units see a much smaller proportion of cases. Therefore, the estimate of incidence may have slightly underestimated the true figure, but it is unlikely to have overestimated it or to be very far from the true value.

The period 2015–2017 was chosen as during this time there was an increase in evidence questioning the effectiveness of surgery and it was before the publication of the national treatment guidelines [4]. Therefore, the authors could report the management occurring in current clinical practice following recent trial data [21]. ESSKA guidelines recommend a non-operative period of 3 months before arthroscopic surgery for certain tear types [5]. The data collection period allowed enough time to factor in periods of non-operative care. However, it is important to note that since 2017 there have been further treatment guidelines, and as a result if the study was repeated the arthroscopy rate may be different from what was reported in this study [4, 5]. A further strength is that two authors independently assessed all MRI reports to ensure the data were accurate. This study is one of the first studies to focus on the language used in MRI reports and how that influences referral to outpatients and subsequent surgery. This has the potential to further inform referrers and also how clinicians produce reports.

Limitations of this work include the absence of important clinical data such as duration of symptoms. As the national guidelines suggest, symptoms in addition to tear patterns play an important role in the management of meniscal tears [4]. The time from the first presentation to time of MRI scan and further review was not reported. Potentially patients could face a delay until the MRI during which time the symptoms could improve with non-operative management or watchful waiting, which may mean that referral is not needed. Future prospective cohort studies are needed to address this deficiency in the future. Additional limitations include the absence of patients who underwent MRI scans in the private sector, therefore, the rate of meniscal tears quoted may be an underestimate. Asymptomatic tears were also not included although the clinical relevance of this group could be questioned.

The results of this study demonstrate the importance of taking into account all clinical features when making treatment decisions. For tear types other than bucket handle tears, it is important to assess the duration and type of symptoms when planning decisions. The authors also advise against radiologists using the term degenerative when describing a meniscal tear as this had no value in subsequent treatment, neither does a recommendation to seek an orthopaedic opinion. For researchers, this study highlights that less than a quarter of patients with a meniscal tear undergo surgery, therefore, when designing studies to assess the strength of an intervention it is important to understand this relates only to a small subset of all patients with a meniscal tear.

## Conclusion

In conclusion, MRI-diagnosed meniscal tears occur more frequently than previously described, at an estimated rate of 222/100,000 cases per head of population. However, only half of these patients present to secondary care and 21% of patients subsequently undergo arthroscopic surgery. Despite arthroscopic meniscectomy being one of the most common knee operations in current practice, there is still a poor understanding of which patients would be best treated with surgery. More research is urgently needed on this topic if consistent, high-quality treatment decisions for patients are to be made.

## Supplementary Information

Below is the link to the electronic supplementary material.

Supplementary file 1 (DOCX 94 KB)
