# Supplementary material for: Meniscal tears are more common than previously identified, however, less than a quarter of people with a tear undergo arthroscopy
Source: Knee Surg Sports Traumatol Arthrosc. 2021 Feb 1;29(11):3892–8. doi: 10.1007/s00167-021-06458-2 (PMC8514344; doi:10.1007/s00167-021-06458-2)
Supplement: Supplementary file 1 — (DOCX 94 KB) [file 167_2021_6458_MOESM1_ESM.docx]

| Baseline feature | Subgroup | Arthroscopy | No Arthroscopy | Probability |
| --- | --- | --- | --- | --- |
| Gender | Male | 203 (23.7%) | 654 (76.3%) |  |
|  | Female | 97 (18.7%) | 421 (81.3%) | 0.031 |
| Age group | 18-40 | 116 (27%) | 313 (73.0%) |  |
|  | 40-50 | 121 (21.2%) | 451 (78.8%) |  |
|  | 50-55 | 63 (16.8%) | 311 (83.2%) | 0.002 |
| Presence of chondral changes | Chondral changes | 131 (19.0%) | 559 (81%) |  |
|  | No chondral changes | 169 (28.9%) | 516 (71.1%) | 0.011 |
| Size of tear | Undisplaced/ small | 17 (13.3%) | 111 (86.7%) |  |
|  | Not undisplaced/ small | 283 (22.7%) | 964 (77.3%) | <0.001 |
| Root tears | Root tear | 11 (32%) | 23 (68%) |  |
|  | No root tear | 289 (22%) | 1052 (78%) | 0.132 |
| Tear type | Bucket handle | 44 (50%) | 44 (50%) | ^a^ |
|  | Complex | 62 (29%) | 152 (71.0%) | ^b^ |
|  | Degenerative | 38 (18.3%) | 170 (81.7%) |  |
|  | Degenerative horizontal/ oblique/ undersurface | 19 (18.8%) | 82 (81.2%) |  |
|  | Horizontal/ oblique/ undersurface | 102 (17.6%) | 475 (82.3%) |  |
|  | Radial | 35 (18.8%) | 152 (81.2%) |  |

Supplementary table 1: A table demonstrating the adjusted analysis exploring the association between baseline variables and arthroscopy.

a – Bucket handle tears had a significantly higher incidence of surgery compared to all other tear types tears (p< 0.001).

b – Complex tears had a significantly higher incidence of surgery compared to degenerative, horizontal/ oblique/ undersurface and radial tears (p< 0.001).

There was no significant difference between all other types of tear patterns.
